# Measuring perinatal complications: methodologic issues related to gestational age

**DOI:** 10.1186/1471-2393-7-18

**Published:** 2007-08-30

**Authors:** Aaron B Caughey

**Affiliations:** 1 University of California, San Francisco; Department of Obstetrics, Gynecology and Reproductive Sciences; San Francisco, California

## Abstract

**Background:**

Perinatal outcomes differ by week of gestational age. However, it appears that how measures to examine these outcomes vary among various studies. The current paper explores how perinatal complications are reported and how they might differ when different denominators, numerators, and comparison groups are utilized.

**Conclusion:**

One issue that can clearly affect absolute rates and trends is how groups of women are categorized by their gestational age. Since most perinatal outcomes can only occur in women and neonates who have delivered, using the number of pregnancies delivered (PD) as the denominator of outcomes is appropriate. However, for an outcome such as antepartum stillbirth, all women who are pregnant at a particular gestational age are at risk. Thus, the denominator should include all ongoing pregnancies (OP). When gestational age is used by week this means using both deliveries during a particular week plus those women who deliver beyond the particular week of gestation in the denominator. Researchers should be careful to make sure they are utilizing the appropriate measure of perinatal complications so they do not report findings that would be misleading to clinicians, patients, and policy makers.

## Background

Traditional perinatal epidemiology utilized metrics such as the neonatal mortality rate (number of neonatal deaths per 1,000 live births) and the perinatal mortality rate (number of neonatal deaths plus stillbirths per 1,000 total births) [1]. Prior to the current age of birth certificate and other large electronic databases, expanding computational power, and statistical software packages, it was recognized that these simple metrics had some small problems, but gave reasonable estimates to compare risk factors. While much of the technological advances have led to better statistical techniques for controlling potential confounders and quicker analysis of large data files, there has been little attempt to develop appropriate metrics to examine rates of perinatal morbidity and mortality and to further improve the accuracy of these measures. This is unfortunate, as the thoughtful approach to measuring complications of pregnancy is paramount and different methods can lead to entirely different outcomes and conclusions.

For example, when examining the complications among all patients with term and postterm pregnancies (37 0/7 weeks and beyond), the denominators can simply be as above, either per 1,000 live births or total births. However, when simply dichotomizing these births to examine the effect of postterm pregnancies (42 0/7 weeks and beyond) as compared to term pregnancies (37 0/7 – 41 6/7 weeks of gestation), the denominator used can matter, depending on the outcome examined. In the setting of antepartum stillbirth, one cannot simply use total births as the denominator because the pregnancies at risk of an antepartum stillbirth are not only those pregnancies delivered at a particular gestational age, but all pregnancies that reached that gestational age (i.e., ongoing pregnancies). Thus, for this simple example, the denominator for the term pregnancies should include all of the term pregnancies as well as the postterm pregnancies, while the denominator for the postterm pregnancies should include just the postterm pregnancies [2]. Most commonly, when describing the risk of complications, the week of gestation is used. Thus, the denominator for an outcome measured with respect to pregnancies delivered, would simply be those pregnancies delivered during that particular week. When the outcome is better examined using ongoing pregnancies, this means using both deliveries during a particular week plus those women who deliver beyond the particular week of gestation in the denominator.

In a recent paper by Joseph [3], the idea of using the ongoing pregnancy denominator was explored beyond simply stillbirths to include all perinatal deaths as well as small for gestational age. There are fundamental problems with modifying the denominator for neonatal deaths and SGA as they can only be diagnosed in neonates that are delivered. Thus, the following text will review different approaches to analyzing a variety of perinatal complications with a focus on determining the correct denominator and identifying the proper control group when making comparisons. Broadly, the discussion will focus on two clinical/research settings: Measuring perinatal complications by gestational age and examining obstetric interventions such as induction of labor.

## Gestational Age

**Figure 1:**
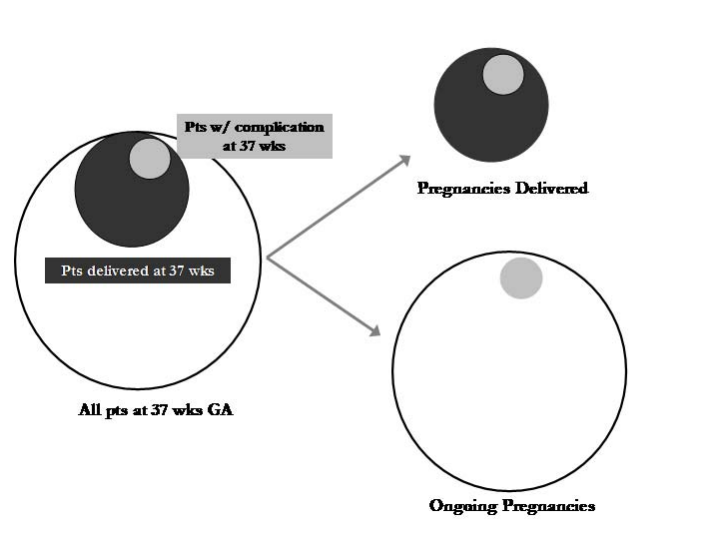
**Comparing the denominators of pregnancies delivered (PD) and ongoing pregnancies (OP)**. Pregnancies delivered consists of just those women delivered during a particular week of gestation. Ongoing pregnancies consists of both those women delivered during a particular week plus those pregnancies that progress beyond a particular gestational age.

As mentioned above, the denominator used is paramount when considering complications of term and postterm pregnancies. The effects of utilizing the wrong denominator in the setting of antepartum stillbirth are primarily: 1) the absolute rate of antepartum stillbirth is increased when the smaller, inappropriate, denominator is utilized (Figure 1); and 2) the relationship of the relative rates of stillbirth by week of gestation is distorted. This problem has been described by several authors [4-6] and was excellently summarized by Dr. Gordon Smith in 2001 [7].

In one of the earlier commentaries on this problem, Yudkin et al [3] noted in 1987 that if the risk of antepartum stillbirth was considered by week of gestation using the denominator of total live births occurring at that gestational age, the risk seemed highest among preterm patients as they had the smallest denominators. However, when they utilized the denominator of ongoing pregnancies, they found that the rate of antepartum stillbirth decreased until 39 weeks of gestation, then increased through 41–42 weeks of gestation. These findings have been reproduced by other authors and using data from a paper published in 2003, the effect of the denominator on antepartum stillbirth is demonstrated (Table 1) [8]. To be more precise, Dr. Smith introduced the idea that since the women who deliver during a particular week are not actually at risk for antepartum stillbirth the entire week, a correction factor can be applied in the formula:

**Table 1:** Antepartum Stillbirth by Week of Gestation Using Different Denominators

**Gestational Age Weeks (n)**	**Using Pregnancies Delivered (#/10,000)**	**Using Ongoing Pregnancies (#/10,000)**
37 (3,964)	27.7	2.4
38 (8,865)	16.9	3.6
39 (13,839)	9.4	4.0
40 (12,456)	4.0	2.6
41 (5,685)	10.6	9.2
>42 (864)	34.7	34.7

PAn = An/[Pn - (0.5 * Bn)]

PAn is the risk of antepartum stillbirth in given week of gestation n

An is the number of antepartum stillbirths occurring during the week of gestation n

Pn is the number of pregnancies which start week n (ongoing pregnancies)

Bn is the number of pregnancies delivered during week n

The importance of this correction factor can be seen in the results of Table 2, which are calculated based on the numbers from the 2001 paper [6]. Similar to Table 1, there is an obvious difference in the overall trend when examining the antepartum stillbirth rates by PD and OP. However, when using the correction factor, the rise in antepartum stillbirths occurs earlier and appears to be more dramatic.

**Table 2:** Antepartum Stillbirth by Week of Gestation Using the Correction Factor for Ongoing Pregnancies

**Gestational Age Weeks (n)**	**Using Pregnancies Delivered (#/10,000)**	**Using Ongoing Pregnancies (#/10,000)**	**Using OP with correction for pregnancies delivered (#/10,000)**
37 (34,189)	74.9	3.7	3.7
38 (89,219)	30.9	4.1	4.4
39 (147,444)	16.9	4.3	4.9
40 (246,467)	11.1	6.4	8.9
41 (146,346)	9.2	7.3	12.1
42 (35,938)	10.3	9.9	19.2
>43 (1,275)	31.4	31.4	62.7

Even the formula above, which is an improvement upon others' work in this field to define the appropriate measure of risk of antepartum stillbirth, has two flaws that can be rectified with better data. The first is that while the use of the correction term will subtract off one half of the births in that particular week in order to get a better estimate of ongoing risk, in any particular week of pregnancy, the number of births per day is unlikely to be a uniform distribution. Rather, like the overall distribution of gestational ages, the number of births will increase prior to 40 weeks of gestation and decrease thereafter. Thus, the exposure to the women delivered at a given week of gestation is likely to be higher prior to 40 weeks of gestation and lower after this threshold. Furthermore, the correction term will lead to a falsely larger denominator for the women prior to 40 weeks of gestation and a smaller denominator for women after 40 weeks of gestation.

The second problem is that while we utilize the day that the patient delivers as the gestational age of the antepartum stillbirth, by definition, the stillbirth may occur prior to the day of delivery. Just how much prior to delivery stillbirth occurs may lead to a lag effect in the difference in the estimated rates. For example, if all of the antepartum stillbirths occurred, on average, a week prior to their delivery, then the rates would necessarily increase a week earlier than currently estimated. Thus, a better approximation of the gestational age of stillbirth would be to generate an estimate of the date at which stillbirth occurs using the date(s) of the last known viable heart rate, last known fetal movement, and the date the stillbirth was documented. Of note, this estimate improves at term when women are often seen at least once a week and are usually asked to report the absence of fetal movement.

Even among those who are interested in improving the antepartum risk assessment of stillbirth, there is disagreement on how to measure and utilize this risk. In a controversial paper on the topic, Cotzias et al used ongoing pregnancy as the denominator, but changed the numerator to include all current and future stillbirths to generate a prospective stillbirth risk [9]. Using this equation, they found that the prospective stillbirth risk was higher with preterm gestations, decreased in term pregnancies until 40 weeks of gestation, then increased through 42 weeks of gestation. They used these calculations to suggest that induction of labor should be considered with the onset of fetal pulmonary maturity at 38 weeks of gestation. The responses to this article found the use of this calculation objectionable as the rate of stillbirth derived from such estimation does not consider the length of time of exposure, nor does the suggestion of early induction of labor consider the costs or the marginal, incremental benefits that might be incurred or achieved [10-12].

While it is clear that ongoing pregnancies should be utilized in the denominator for antepartum stillbirth as these are the women at risk, it is less clear whether other maternal or neonatal complications of pregnancy should be considered in the same way. Consider neonatal and perinatal deaths (stillbirths plus neonatal deaths). In order for a neonatal death to occur, the fetus had to become a neonate by being born. Thus, the fetuses of ongoing pregnancies are not yet at risk. This leads to the rate that has been used historically, that is, the number of neonatal deaths divided by the number of live births [6]. Therefore, this is a problem with the metric proposed by Joseph [3]. In a recent paper which suggests combining stillbirths plus neonatal deaths into one outcome, it is demonstrated that such a metric finds the risk of perinatal death to rise beyond 35 weeks of gestation, supporting possibly earlier induction of labor. Unfortunately, while at term, such decision-making based on the ongoing risk of stillbirth is sensible, causing more preterm births which are themselves associated with higher rates of morbidity and mortality may not be supported by such algebraic manipulation. A better measure of the total ongoing risk to a pregnancy was proposed by Smith as what he describes as the cumulative probability of perinatal death which combined both stillbirth and neonatal deaths [7].

What about measures of neonatal morbidity? These might include five-minute Apgar scores less than 4 or 7, admission to the neonatal intensive care unit, birth trauma, or intrauterine growth restriction (birthweight < 3rd, 5th, or 10th percentile). For all of these outcomes, the neonate cannot experience the outcome until birth, so the standard measure utilizing pregnancies delivered should apply. The birthweight metric should also be examined using pregnancies delivered, since one does not know the actual birthweight until the infant is delivered. Interestingly, one could use the estimated fetal weight from sonographically predicted birthweight to generate risks of intrauterine growth restriction. If one did perform weekly ultrasounds of an entire population of pregnant women, then, and only then, could ongoing pregnancy be used as the denominator.

Again, Joseph uses "revealed SGA" as the number of SGA fetuses born at a particular gestational age over a denominator of all ongoing pregnancies [3]. This metric, will of course increase over time as the denominator shrinks with further deliveries, so again provides little insight to an optimal time of delivery. The only types of neonatal morbidities that can be measured using ongoing pregnancies as a denominator would be those that can be measured in all pregnancies. For example, if we routinely measured all in utero fetuses by ultrasound and identified the SGA fetuses, then the proper denominator would be ongoing pregnancies. However, since we wouldn't deliver these SGA fetuses before 34–36 weeks of GA, the numerator would have to be the SGA fetuses identified by ultrasound, not delivered.

For maternal complications of pregnancy, it is more difficult to identify the group of women at risk for various complications of pregnancy. There are some complications which clearly occur in women who are antepartum (Table 3). For example, preeclampsia is usually an antepartum diagnosis. Thus, it makes sense to utilize ongoing pregnancies as the denominator when estimating the risk of preeclampsia at a particular week of gestation [2]. For mode of delivery (cesarean, vaginal, or operative vaginal) and outcomes related to the mode of delivery such as wound complications or perineal lacerations, clearly pregnancies delivered should be used as the denominator. However, there are a number of complications that can occur either prior to the onset of labor or during labor such that determination of the appropriate denominator to be used may be difficult. For example, chorioamnionitis occasionally occurs prior to the onset of labor. In this setting, its risk should be determined based on all women at risk, i.e., ongoing pregnancies. However, the majority of chorioamnionitis occurs during labor, and thus would only apply to the pregnancies delivered during a particular week of gestation. The same holds true for placental abruption, which can occur prior to the onset of labor, but its risk is increased during labor. When attempting to describe these risks, it is important to specify what aspect of the complication is being examined.

**Table 3:** Appropriate Denominator* when Considering Pregnancy Outcomes and Complications by Week of Gestation

Pregnancies Delivered (PD)	Mixed	Ongoing Pregnancies (OP)
Forceps delivery	Antepartum stillbirth	Abruption
Vacuum delivery	Oligohydramnios (if measured)	Chorioamnionitis
Epidural use	Preeclampsia/Eclampsia	Primary cesarean
3^rd ^and 4^th ^degree lacerations	Onset of labor	Uterine rupture
Cesarean in labor	Antepartum hemorrhage	
Postpartum hemorrhage	Premature rupture of membranes	
Shoulder dystocia	Preterm birth	
Neonatal outcomes		

*The type of denominator denotes the population at risk for the outcome or complication

Of note, in studies that examine perinatal morbidity by gestational age at term, a number of complications appear to increase with increasing week of gestation beyond 38 to 39 weeks. Complications which do increase by week of gestation at term include neonatal outcomes such as acidemia and macrosomia as well as maternal morbidities such as preeclampsia, postpartum hemorrhage, perinatal infection, and cesarean delivery [2,7,13-16].

## Induction of Labor and Cesarean Delivery

For some time, it has been assumed that induction of labor is associated with an increased risk of cesarean delivery [17] Interestingly, the majority of studies reporting that labor induction is associated with an increased rate of cesarean delivery are not randomized trials [18-20]. However, when induction of labor and mode of delivery are considered more carefully, some interesting contradictions arise. While large, prospective, randomized, controlled trials of labor induction in low-risk women at term are yet to be conducted, a number of randomized trials which examined induction of labor in several high-risk subgroups, including post-term pregnancy, pregnancies complicated by diabetes, and pregnancies suspicious for large-for-gestational age fetuses. Studies that examined pregnancies at or beyond 41 weeks of gestation have demonstrated a decrease in cesarean delivery among women who have undergone induction of labor [21,22]. Prospective trials report no statistically significant difference in the rate of cesarean delivery in pregnant women with diabetes [23] and in those who were induced for suspected fetal macrosomia [24].

**Figure 2:**
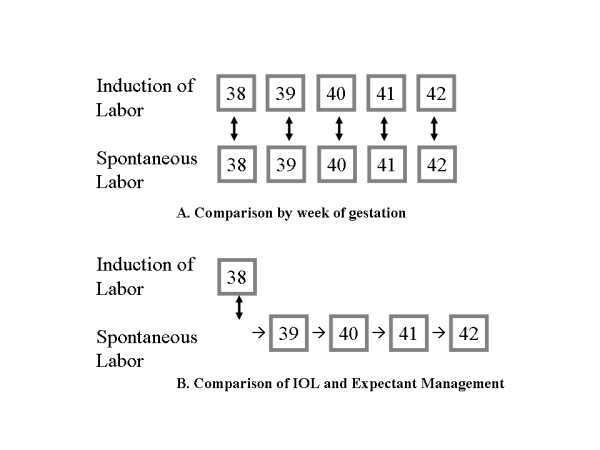
A – Traditionally, when controlling for gestational age, one compares women induced at a given gestational age to those who experience spontaneous labor at the same gestational age. B – Clinically, we are deciding between induction of labor at the current gestational age versus expectant management leading to delivery at a greater gestational age [24].

Thus, the existing prospective randomized trials directly contradict the retrospective or prospective cohort or case-control studies by demonstrating either a decrease or no difference in cesarean delivery. One possible reason for the discrepancy between these two types of studies is that the majority of the cohort and case-control studies do not control for gestational age. Since induction of labor is likely to occur more often as gestational age increases, and increasing gestational age itself is a risk factor for cesarean delivery [15], this is an important confounder to consider in the analysis of such data. However, confounding by gestational age does not entirely explain the above discrepancy, since there are recent studies that do control for gestational age and still find that induction of labor is associated with cesarean delivery [17,18]. Consider the analytic design: by either matching on gestational age or utilizing multivariable techniques to do so, one makes a comparison between women who are induced at a given gestational age as opposed to women who experience spontaneous labor at the same gestational age (Figure 2A). However, when caring for a pregnant woman at term, a clinician's options are not between induction of labor and spontaneous labor; rather, the options at hand are induction of labor versus expectant management of the pregnancy, the latter of which results in either spontaneous labor or induction at a greater gestational age (Figure 2B).

In order to examine the effect of comparing induction of labor to spontaneous labor by week of gestation, a recent analysis of this question was conducted [25]. Induction of labor at 38, 39, 40, and 41 weeks of gestation was compared to expectant management beyond each of the gestational ages, respectively. The expectant management group, similar to the induction group, excluded women who underwent a cesarean delivery prior to the onset of labor. Similar comparison variables were created for 39, 40, and 41 weeks of gestation.

Induction of labor was first compared to expectant management with cesarean delivery as the primary outcome of interest. Induction of labor was then compared to spontaneous labor at each gestational age to demonstrate the difference between these two types of comparisons. In the comparison that is commonly made, namely when women who underwent labor induction were compared to those with spontaneous labor, the cesarean delivery rate was higher for women being induced at each gestational age (Table 4). However, when women who underwent labor induction were compared to the expectant management group (i.e., all women who ultimately delivered at a greater gestational age), the differences in the bivariate comparisons were not statistically significantly different. Further, when the potential confounders were controlled for, the cesarean delivery rate was statistically significantly higher for the expectant management group at 38, 39, and 40 weeks of gestation with odds ratios ranging from 1.80 (95%CI: 1.29–2.53) at 38 weeks to 1.27 (95%CI: 1.00–1.62) at 40 weeks of gestation.

**Table 4:** Induction of Labor Compared to Expectant Management

**Week of Induction**	**IOL CD**	**Expt mgmt CD**	**AOR (95% CI)†**	**Spontaneous Labor CD**
38 weeks	11.9%	13.3%	1.80 (1.29 – 2.53)	7.0%*
39 weeks	14.3%	15.0%	1.39 (1.08 – 1.80)	9.1%*
40 weeks	20.4%	19.0%	1.27 (1.00 – 1.62)	10.9%*
41 weeks	24.3%	26.0%	1.26 (0.99 – 1.61)	14.9%*

CD = Cesarean Delivery, Expt Mgmt group includes all women delivered beyond the particular gestational age of the induction of labor comparison group

† Multivariate comparison of cesarean delivery rates between the induction group and the expectant management group. An AOR > 1 implies higher cesarean rates among the expectant management group.

* p-value < 0.01 when compared to IOL at same GA

Multivariate analysis controlling for parity, maternal ethnicity, age, BMI, insurance, education, preeclampsia, diabetes, epidural use, and year of delivery

Thus, it appears that in this setting, choosing the appropriate comparison group is enormously important as it changes the findings from induction of labor leading to higher rates of cesarean delivery to induction of labor leading to either no difference or even lower rates of cesarean, depending on the gestational age. These findings are less surprising, given the recent retrospective study which demonstrated that induction of labor may be used to decrease cesarean birth [26].

## Conclusion

With the increasing availability of large birth cohorts and datasets, it is incumbent upon researchers to carefully choose their metrics and comparison groups when examining perinatal complications and mode of delivery. In both of the settings described above, determining the wrong absolute rate, or more seriously, concluding findings in the opposite direction, could lead to misleading information for clinicians, patients, and policy-makers alike. In this light, it is also important for journal editors and reviewers to ensure that appropriate measures are being utilized.

In Joseph's paper [3] it should be noted that the intent of combining stillbirths plus neonatal deaths as a measure of pregnancy outcome for interventional trial is well-intended. However, how best to do so is a bit unclear. If a trial was investigating a drug utilized throughout pregnancy and the intent was to have mortality play a role as one of the outcomes, one simple way would be to simple compare the proportions of pregnancies which experienced a mortality event, whether fetal or neonatal. However, if the intent was to develop a metric which incorporated weeks of pregnancy at risk, it should be made clear that this would really only apply to stillbirths, not neonatal deaths. In that setting, one could simply control for gestational age at delivery, or use as a denominator, total weeks of pregnancy for the stillbirths, but the neonatal deaths would need to be examined as a separate outcome. Certainly, the metric of revealed SGA would, similarly, be best as proportion of SGA infants out of the total.

When the issue of outcome by week of gestation is the primary outcome to be examined, careful choice of the appropriate denominator is paramount as demonstrated both in the work on stillbirth and induction of labor [7,25] However, in the setting of interventional trials, this is less important. Certainly, the most important issue is that for any study, careful attention be paid to choosing the appropriate outcome measures and how best to measure them.

## Statement of Competing interests

The author(s) declare that they have no competing interests.

## Pre-publication history

The pre-publication history for this paper can be accessed here:


